# Cold Snare Polypectomy in Pediatric Polyposis: A Multicenter Experience

**DOI:** 10.3390/children12030291

**Published:** 2025-02-26

**Authors:** Hunter J. Friesen, Thomas M. Attard, Andrew Y. J. Liman, Osamu W. Yasui, Catharine M. Walsh, Roberto Gugig, Monique T. Barakat

**Affiliations:** 1 Division of Gastroenterology, Hepatology and Nutrition, Children’s Mercy Hospital Kansas City, Kansas City, MO 64108, USA; 2 Department of Pediatrics, University of Missouri-Kansas City School of Medicine, Kansas City, MO 64108, USA; 3 Division of Pediatric Gastroenterology, Lucille Packard Children’s Hospital, Stanford University Medical Center, Stanford, CA 94304, USA; 4 Division of Gastroenterology, Hepatology and Nutrition and the Research and Learning Institutes, The Hospital for Sick Children, Toronto, ON M5G 1E8, Canada; 5 Department of Paediatrics and the Wilson Centre, University of Toronto, Toronto, ON M5T 3M6, Canada

**Keywords:** cold snare polypectomy, pediatric polyposis, endoscopic resection, multicenter study

## Abstract

**Background**: Cold snare polypectomy (CSP) is a well-established and recommended technique in adult gastroenterology for the safe, efficient and complete removal of nonpedunculated lesions up to 10 mm, with piecemeal excision possible for larger lesions. However, the application of CSP in pediatric patients remains underexplored. This study summarizes a multicenter experience of CSP in pediatric polyposis patients, focusing on safety, efficacy and clinical outcomes. **Methods**: This retrospective study was conducted at two pediatric tertiary centers, encompassing patients aged 1 to 21 years with polyposis who underwent colonoscopy with CSP and hot snare polypectomy (HSP) between January 2022 and January 2023. Patient demographics, procedure characteristics, polyp details and clinical outcomes were analyzed. **Results**: A total of 477 CSPs were performed in 63 colonoscopies. Satisfactory bowel preparation was noted in 79% of procedures, with a pooled mean procedure duration of 52 min and cecal intubation achieved in 98%. Polyps resected by CSP ranged from 3 to 70 mm in size and were predominantly left-sided. Tissue retrieval was complete in 94% of cases and partial in 5%. Mild intraprocedural bleeding occurred in 25% of CSP cases, requiring endoclip placement in 19%, with no post-procedural bleeding or significant complications observed. Comparatively, CSP demonstrated favorable bleeding rates relative to HSP. At two-week follow-up, four patients required emergency evaluation for unrelated complaints, but with no adverse events attributed to CSP. **Conclusions**: CSP is a safe and effective technique for the removal of sessile polyps in pediatric patients with polyposis. Mild intraprocedural bleeding, when observed, was effectively managed with standard hemostatic techniques. These findings support the potential of CSP as a preferred modality for sessile polyp removal in pediatric patients, though further research is warranted to define its role across broader pediatric populations and practice settings.

## 1. Introduction

Gastrointestinal, predominantly colonic, polyps are routinely encountered in pediatric gastrointestinal patients, often presenting with bleeding, extrusion and rarely with anemia or obstruction. They occur in about 6% of children undergoing colonoscopy [1]. A subset of syndromic patients can harbor numerous, including extracolonic, polyps and may entail risk of malignant transformation. The most frequently encountered hereditary polyposis syndromes include Familial Adenomatous Polyposis (FAP) (1:8,000–10,000), Peutz–Jeghers Syndrome (PJS) (1:25,000–300,000) and Juvenile Polyposis Syndrome (JPS) (1:100,000). Endoscopic polypectomy is incorporated in the management guidelines for this population of patients [2].

Management of hereditary polyposis syndromes starts in early childhood and adolescence, includes regular surveillance colonoscopy and may include surgery in the second decade of life. These patients are transitioned to adult provider care between the ages of 18 and 21 years in the United States, with the specific timing and process of transition of care coordinated to minimize disruption of care [3]. The established, traditional approach to polypectomy in pediatric patients is centered around hot snare polypectomy (HSP). When specified, most published sources describe HSP as the sole technique [4,5,6], with refinements including stalk injection, endoclip application or endoloop-assisted strangulation, when required [7]. More recently, endomucosal resection has been introduced following its well-defined role in adult gastroenterology practice [8]. Cautery and coagulation-based techniques, however, have significant limitations, including delayed post-polypectomy bleeding (DPPB) in 3–4% of procedures, with larger (6–10 mm and >20 mm), right-sided lesions, multiple polyps and sessile polyps being at greater risk [9,10]. In addition, HSP entails the risk of post-polypectomy electrocoagulation syndrome (PPES) presenting with abdominal pain and fever several days after HSP in 1–3% of patients, including children [11,12]. Rarely, PPES can evolve into post-polypectomy perforation [13]. Cold snare Polypectomy (CSP) has been discussed as an option for children with sessile polyps up to about 5–7 mm [14].

CSP is well entrenched in adult gastroenterology practice and the technique has been exhaustively described [15]. A safety study of CSP reported that lesions measuring 6–9 mm could be resected without increasing the bleeding rate [16]. CSP is superior to cold forceps polypectomy (CFP) for diminutive polyps (≤5 mm) and is associated with a lower rate of DPPB, lower risk of major bleeding and equivalent completeness of resection and polyp recurrence risk compared with HSP in polyps <20 mm in size [17,18,19]. CSP is identified as first choice for treating colorectal polyps ≤9 mm by the 2017 European Society of Gastrointestinal Endoscopy Guidelines [20], the 2020 US Multi-Society Task Force on Colorectal Cancer Guidelines [21] and the 2022 Japan Gastroenterological Endoscopy Society Guidelines [22]. Refinements to the CSP technique include cold snare EMR with submucosal injection [23] and underwater CSP [24].

The pediatric polyposis population presents opportunities and challenges in the adoption of CSP as a standard technique, potentially preferred over HSP for sessile lesions. The reduction in post polypectomy bleeding, elimination of delayed electrocautery syndrome and increased efficiency with equivalent or superior complete resection rates are potential advantages; especially in the context of the thinner intestinal wall in the younger patient population [25]. The wider adoption of CSP presents the challenge of additional training requirements, trailing the experience in adult gastroenterology, so this technique’s applicability must be critically appraised.

There is a paucity in the literature relating to CSP in the pediatric population despite anecdotal evidence that it is widely accepted as a technique by pediatric advanced endoscopists in tertiary referral centers. Indeed, there are no published studies relating to the safety, efficacy and outcomes of CSP in pediatric patients.

Herein we describe the experience of two pediatric tertiary centers with advanced endoscopy expertise, including CSP technique. The goal of this study was to assess the safety and effectiveness of CSP in pediatric patients with polyposis toward potentially establishing CSP as the standard of care for polypectomy in a subset of pediatric polyposis patients.

## 2. Methods

- Study Design and Setting

This multi-center parallel retrospective study evaluating polyp resection practices was conducted at both Children’s Mercy Hospital (CMH) and Lucile Packard Children’s Hospital (LPCH), Stanford, between 1/2022 and 1/2023. Both centers are high-volume tertiary care pediatric academic medical centers.

- Patient Selection and Population

Data from pediatric patients (≤21 years of age at the time of the procedure) who underwent colonoscopy with polyp resection were included and analyzed. The cohort from CMH included only patients followed through the Hereditary Polyposis Clinic at that institution and who were identified on screening chart review as having undergone CSP. At LPCH, all patients who met the age criteria and underwent polypectomy during the study period were included in this analysis.

- Procedure Details

Colonoscopy preparation protocols included age-specific regimens of clear liquid diets, hydration encouragement, and incremental dosing of PEG solution (with or without Senna/Bisacodyl for older children) to achieve clear or light-yellow stools prior to the procedure. Informed consent was obtained from the parent or legal guardian for children under the age of 18 at the time of the procedure, and from the patient if 18 or older. Intraoperative sedation was managed by a pediatric anesthesiologist and used to achieve general anesthesia with standard ASA monitoring (ECG, SPO_2_, BP, RR, Temp) being used in accordance with ASA guidelines [26]. The specific anesthetic technique varied based on age, medical history, and patient preference but could include midazolam preoperatively for anxiolysis. Induction of anesthesia was with either lidocaine 1–2 mg per kg and propofol 2–3 mg/kg or a mask induction with sevoflurane/nitrous with IV placed after induction. Natural airway, laryngeal mask airway (LMA) or endotracheal intubation was tailored to patient needs. Maintenance of anesthesia was with either a propofol infusion 200–300 mcg/kg/min or sevoflurane to 1MAC.

**Figure 1:**
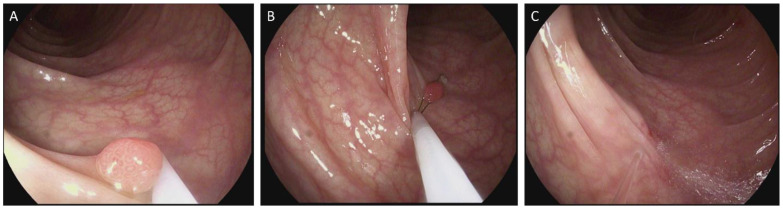
Transverse colon polyp in a 14-year-old with JPS resected with CSP. (**A**) Polyp is visualized and brought to 6 o’clock position; size is estimated by placing dedicated Cold Snare (Exacto^®,^ Steris, Mentor, OH, USA); external sheath diameter 2.4 mm) abutting polyp surface. (**B**) The snare is opened to slightly greater than the size of the polyp to ensure that a rim of normal tissue is resected, and is placed over the polyp, parallel to the colon wall followed by closure of the snare, with (**C**) post resection irrigation and inspection for lesion margins, completeness of resection, bleeding or evidence of perforation.

Polyp resection by cold snare was accomplished using either Exacto (Steris, Mentor, OH, USA) or Sensation (Boston Scientific, Marlborough, MA, USA) snares. CSP involved the following steps, as described elsewhere (Figure 1) [27]: (1) The tip of the endoscope was positioned so that the polyp was easily accessible, ideally at the 6 o’clock position; (2) the snare catheter was advanced to the lesion to be resected; (3) the snare was opened to grasp polyp tissue within the snare; (4) the snare was closed steadily and rapidly to cut through tissue surrounding the polyp; (5) polyp tissue was recovered by suction or Roth net if larger than suction could accommodate, as described below. (6) The resection site was inspected to evaluate completeness of resection and for bleeding. Non-pedunculated polyps over 35 mm were raised with saline prior to snare resection. A total of 12 polyps (from LPCH) met these criteria and were raised for resection. This is an established technique for the resection of polyps in this size range.

Both centers practice a technique of suctioning resected polyps into a suction polyp trap (Steris^®^, Mentor, OH, USA); immediately after resection and once more after lavage of the segment of bowel. The polyp trap has different compartments that are rotated in accordance with the segment of intestine being operated upon. The polyp trap was emptied and cleaned once all compartments had been used and the number of resected specimens was counted, recorded and reconciled with the number of polypectomies performed in that segment and submitted for histopathologic evaluation. For polyps that were too large to be suctioned into the suction/biopsy channel of the colonoscope, a Roth net was used to retrieve the polyp. The histopathologic report details both the histologic subtype of the resected polyp as well as any concerns on completeness of resection.

- Data Collection and Variables Analyzed

The data were collected by 4 researchers (2 LPCH, 2 CMH). This study was conducted as part of quality analysis through a review of our electronic endoscopy records and entry of the relevant information into the datasheet. Variables abstracted and analyzed included: patient age, demographics, size and number of polyps resected, diagnosis of patient (if polyposis syndrome), modality of polyp resection, polyp type as determined by the pathology report from polyps resected, adverse events associated with polyp resection and management of these adverse events.

Data from each center were systematically collected in a retrospective manner, and then fully de-identified single center data were preliminarily analyzed at each center. The results were compared between the two institutions and aggregated for this study.

We recorded the occurrence of any adverse event. Adverse events were classified by event type (significant bleeding, perforation, cardiopulmonary event associated with anesthesia or other event as specified). Minor oozing/bleeding noted intra-procedure that was observed until resolution or managed by clip placement in the same session as polyp resection does not reflect a true adverse event, but rather was recorded to fulfill one of the parameters of our quality analysis efforts for assessment of CSP. This categorization of intra-operatively managed bleeding as a part of resection rather than an adverse event is in alignment with the endoscopy society consensus classifications of adverse events [28]. Adverse events were determined by retrospective review of the electronic medical record and anesthesia documentation, as well as follow-up calls/evaluations that are routinely conducted post-procedure.

- Statistical Analysis

Data were analyzed using descriptive statistics, with continuous variables summarized using means and standard deviations and categorical variables summarized using proportions. Intraprocedural bleeding rates with CSP and HSP were compared with Fisher’s exact test and statistical significance was defined as two-tailed *p* < 0.05.

## 3. Results

- Patient and Procedure Characteristics

A total of 63 procedures were analyzed, 34 from Center A (CMH) and 29 from Center B (LPCH). The mean age of patients was 14.3 years overall, with an age range of 4 years to 21 years. In total, 50/63 patients (79%) had a Boston Bowel Preparation Score (BBPS) greater than 6 (acceptable) for the procedure during which polyps were resected. The one patient with poor prep that precluded successful cecal intubation and polypectomy was rescheduled to the next available date for the procedure. A trainee was present for 31/63 procedures (49%). The pooled mean procedure duration was 52 min. Cecal intubation was achieved in 62/63 (98%) of patients. Table 1 depicts these demographic and outcome measures.

- Polypectomy Characteristics

The mean number of polyps resected per procedure was 12.5. HSP and CSP were performed in 38 and 81% of procedures, respectively. CSP was performed on 477 lesions (mean 10.3/procedure) with a size range of 3–70 mm. CSP includes cold endoscopic mucosal resection, which was performed for polyps >35 mm in size. When recorded, polyp distribution for polyps resected by CSP was notable for 25% in the right colon, 9% in transverse colon and 40% in left colon at Center A (CMH) and 32% in the right colon, 17% in the transverse colon and 51% in the left colon at Center B (LPCH). Tissue retrieval was reported as good at both sites; complete 91% [31/34] or partial 6% [2/34] at Site A (CMH), and complete 97% [28/29] or partial 3% [1/29] at Site B (LPCH).

- Intraprocedural Bleeding and Post-Procedural Adverse Events

Intraprocedural bleeding was noted in 29% (16/54) and required intervention (endoclip placement) in 22% (12/54). The odds ratio for intraprocedural bleeding was 0.13 for polyps treated with CSP compared to those treated with HSP without variable risk of bleeding based on size of polyps resected during the colonoscopy procedure (95% confidence interval: 0.03–0.57). No post-procedure bleeding or related complications were reported. However, at 2-week follow-up, four patients were directed for Emergency Department (ED) evaluation but for apparently unrelated complaints, including intra-procedurally placed video capsule retention. At LPCH, two CSP patients were seen in the ED for post-procedure abdominal pain; imaging was reassuring and pain self-resolved but one of these patients was admitted for observation. This is compared with five non-CSP patients who presented to the ED post-procedure; three had post-procedure bleeding, two required observation and one required repeat colonoscopy for hemostasis.

## 4. Discussion

CSP has been widely adopted in adult gastroenterology and included in treatment guidelines, though its adaptation to the pediatric population requires a thorough evaluation. This is the first study to systematically study the feasibility, safety and outcomes of CSP as a standard technique for the removal of colorectal polyps in the pediatric population.

- Comparison with Adult CSP Data and Implications for Pediatric Use

The safety and efficacy of CSP is well established in adult populations and represents the current standard of care for resection of nonpedunculated polyps less than 10 mm in diameter [15]. CSP with submucosal lift—a ‘cold EMR’ approach—is also very commonly used for the resection of larger polyps to avoid potential thermal injury, avoid cautery artifact of the specimen and to minimize occurrence of delayed bleeding, and in our study this technique was used for polyps over 35 mm in size [8,15,21,29]. In our study, long term outcomes were not tracked; however, in adults, several studies have demonstrated that CSP is effective for the complete resection of colorectal polyps with low recurrence rates comparable to hot snare polypectomy [17]. Although these observations are encouraging, there are important differences in polyp histology and underlying disease processes in children compared with adults that require further study of the long-term effectiveness of CSP in children.

Our multi-center retrospective evaluation of pediatric polyp resections supports the safety of CSP in this population. The size range of resected pediatric polyps, 3 to 70 mm, was broad, contributing to a more robust evaluation of safety and efficacy. Based on these results, the institutions conducting this study have transitioned to exclusive CSP for the resection of sessile polyps under 10 mm in size in pediatric patients. Endoscopic resection of larger sessile polyps in pediatric patients at our institutions is also now commonly accomplished using submucosal lift and cold snare resection. CSP may also be considered for semi-pedunculated and short pedunculated lesions under 10 mm. For these semi-pedunculated and short pedunculated lesions with narrow and short stalks, it may be appropriate to prioritize minimizing wall injury over immediate oozing, which can be managed by endoclip placement prior to resection to eliminate blood flow through the dominant vessel in the polyp stalk or immediately following resection to achieve hemostasis and prevent ongoing bleeding. More studies on prophylactic endoclip placement approaches prior to CSP in these lesions are needed before firm recommendations can be made.

CSP should be considered in pediatric patients, especially when other size differences relative to adult patients are considered, including thickness of the submucosal space, width of the bowel lumen and available operating space. Use of cold snare-based approaches is expected to eliminate thermal injury during polyp resection given the intrinsic omission of electrocautery [30]. Furthermore, without the cautery of a hot snare, any bleeding source is more likely to be recognized and treated during polyp resection rather than resulting in a delayed post-polypectomy bleed that would require a repeat colonoscopy for hemostasis. While it is not possible to definitively ‘prove’ the above for pediatric patients, there is an abundance of evidence and organization-level recommendations supporting CSP for sessile polyp resection in adult patients [15,20,21,22]. Coupled with the potential unique benefits of CSP in children, we believe it is reasonable for institutions to consider shifting standard practice toward CSP for the resection of sessile polyps under 10 mm in size.

- Safety and Efficacy of CSP in Pediatric Patients

Data from this study support the safety and efficacy of CSP in pediatric patients, particularly the absence of significant intra-procedural and major post-procedural adverse events, which is a significant finding. Intra-procedure bleeding will occasionally be observed with either CSP or HSP, but in our study we observed significantly lower bleeding rates in polyps treated with CSP compared to HSP (odds ratio 0.13, *p*-value 0.018). This effect is likely exaggerated by confounding; specifically, larger polyps are more likely to bleed [31], and more likely to be treated by HSP. Future randomized controlled trials would help to reduce confounding from this phenomenon.

Management of intraprocedural bleeding varies based on bleeding source, characteristics and location. In this study, a subset of resected polyps at both institutions were notable for some instances of post-resection bleeding that was recognized during the procedure. At CMH, three of these seven instances resulted in clip placement for hemostasis. At LPCH, clip placement was performed whenever even a small amount of post-resection bleeding was recognized. This difference in practice underscores the lack of, and need for further studies to determine the indications for and optimal application of secondary hemostasis. Importantly, none of these patients had delayed bleeding following colonoscopy. These data suggest that, as for adult patients, CSP could be a preferable option for pediatric patients.

- Impact of Procedural Factors

Bowel preparation was satisfactory in 77% of patients; however, for those with extremely poor bowel preparation (solid stool that could not be cleared), this impaired visualization and prevented cecal intubation. This was not an issue for most patients, and cecal intubation was achieved in 98% of patients. Thus, while CSP, like any polypectomy approach, can be limited by bowel preparation [2], this was not a substantial issue in the present study. The pooled mean duration of the procedure was 53 min, which is longer than for routine diagnostic colonoscopy, but this is expected based on the number of polyps resected in each procedure (average of 11 polyps resected per procedure at LPCH, for example). Furthermore, the presence of a trainee significantly increased the duration of the procedure. This is wholly anticipated given that procedure efficiency improves with experience [32]. In our institutions, whenever possible, this is discussed and planned for ahead of the actual procedure so that the additional focus on teaching, demonstration and supervision can take place with predetermined goals.

In our study, we observed no significant post procedure adverse effects attributable to CSP. Hemostatic measures were utilized intra-procedurally in a subset of patients, but as part of the resection technique itself, and not as a response to an adverse event. In all such instances, bleeding was controlled through endoclip placement and no subsequent adverse events were noted. This mirrors the extensive published experience in adult gastroenterology. Furthermore, no instances of PPES were noted, as would be expected given the association with electrocautery [30]. Overall, our observations suggest that CSP is safer than HSP in a broad range of colorectal sessile polyposis.

Polypectomy, including CSP, requires good or excellent bowel preparation [2]. This is a prerequisite for optimal visualization, especially of small lesions, as well as necessary toward minimizing contamination in the event of perforation. Operator characteristics include a high cecal intubation rate and the ability to reduce colonoscope looping ahead of polypectomy. Staff assisting in polypectomy need to be experienced in the different techniques, and pre-procedural preparation requires adequate accessory items including a range of snare sizes along with the full range of hemostasis tools being readily available.

We report on a broad range of polyp sizes amenable to CSP, with most being sessile and 10 mm or under. Larger sessile polyps were resected with combined submucosal lift and CSP, using a cold EMR approach, which has been found to be as safe and effective as CSP for the resection of larger polyps [29,33].

Both centers in our study used a dedicated, thin CSP device, based on published reports on optimizing tissue retrieval, likely related to the thinner accessory diameter [34]. The likelihood of bleeding is reported as similar to that with HSP snares [35]. In our experience, however, the thickness of the HSP device renders the ideal placement of the loop around the lesion technically more difficult.

In our study, whenever recorded, tissue retrieval tallied well with polypectomy count; there were no concerns of incomplete resection reported. This aligns with reported observations in earlier studies in adults suggesting identical high retrieval rates, and shorter procedure times [34]. Nonetheless, a proportion of polyps are, unfortunately, not retrieved in colonoscopy, particularly when bowel preparation is not optimal. Some studies support that only the largest polyps need to be sent for pathological analysis [36]. The larger polyps are typically retrieved, while it is the diminutive, resected polyps that are not retrieved.

- Training in CSP

Training in CSP is not currently a requirement in pediatric gastroenterology fellowship programs [37] and most practicing pediatric gastroenterologists are not performing the technique. This results in both inadequate exposure to CSP during training and a lack of expertise required to teach it. To address this, there is a pressing need for continuing professional development programs to upskill faculty, ensuring they possess the necessary skills to perform and teach CSP effectively. Adult studies have demonstrated a long learning curve for CSP [38], with most trainees not achieving competency by the end of fellowship. High rates of incomplete resections by practicing gastroenterologists highlight the dependence of polypectomy effectiveness on operator technique [39,40]. Given CSP is a distinct skillset, there is a need for standardized curricula to ensure the technique is consistently taught, practiced and assessed using validated tools, such as the Cold Snare Polypectomy Assessment Tool [38,41]. Additionally, effective simulation models and mastery-based curricula will be essential to facilitate CSP training, enabling deliberate practice with feedback in a controlled environment, and providing the high volume of polypectomies needed to achieve competence [42,43]. Addressing these gaps will enable pediatric endoscopy training to incorporate CSP more effectively, ensuring that both trainees and faculty are equipped with the required skillset to perform the technique safely and proficiently.

- Limitations of the Study

Our study has limitations inherent to retrospective design, including incomplete data, confounding and unstructured recording of polyp characteristics inherent to the procedure documentation software in use at both centers (Provation^®^, Fortive Corporation, Everett, WA, USA. Indeed, the underlying rationale for the quality improvement effort is to improve documentation. Complete recovery and accurate localization of resected polyps is especially difficult in the context of multiple polypectomies, as in this study. The sample size may not be robust enough to elicit rare but serious adverse events such as perforation, and observations in this cohort may not be generalizable to the broader population of endoscopists. Data regarding long-term follow up were also not available, precluding conclusions about the long-term outcomes associated with CSP. These limitations can be mitigated in future prospective, multicenter studies, validating these findings across diverse pediatric populations and practice settings.

## 5. Conclusions

Our observations suggest that CSP is a safe and effective technique in pediatric polyposis. It is the preferred modality for the excision of sessile polyps under 10 mm in size. It decreases the likelihood of bleeding complications precluding PPES, and therefore delayed perforation. We recommend CSP to remove sessile polyps under 10 mm in diameter in children, with endoclip application for observed bleeding. As with other techniques, pre-procedural preparation including snare selection, training of proceduralists and staff, and clear communication regarding post-operative care, are key considerations. Further research is needed to define more clearly the role of CSP in different subsets of pediatric patients, broadening the population to sporadic polyps or those with larger or more difficult-to-reach polyps. More robust evidence is needed to support the inclusion of CSP in future pediatric guidelines.

## Figures and Tables

**Table 1:** Pooled analysis of procedure, polypectomy and cold snare polypectomy characteristics.

	Center A (CMH)	Center B (LPCH)	Combined
Total procedures involving CSP	34	29	63
**Procedure Characteristics**			
Mean age in years (range)	13.7 (4–19)	15.2 (4–21)	14.4 (4–21)
BBPS score ≥ 6 (%)	30 (88)	20 (69)	50 (79)
Trainee present (%)	13 (38)	18 (62)	31 (49)
Mean duration in minutes (SD)	65.3 (27.1)	38 (13)	52.3
Cecum intubated (%)	34 (100)	28 (97) *	62 (98)
**Polypectomy Characteristics**			
Mean polyps removed per procedure (CSP + HSP)	13.2 †	11 (range 1–36)	12.5
HSP (% of cases)	8 (24)	16 (55)	24 (38)
CSP (% of cases)	33 (97)	18 (62)	51 (81)
Polyp size range in mm	3–60	N/A	N/A
**CSP Procedures Characteristics**			
Polypectomy # (mean CSP/pt) ◊	281 (12.7)	196 (6.8)	477 (10.3)
Polyp size range in mm	3–20	4–70 **	3–70
Polyp localization Right (including cecum) Transverse Left (including rectosigmoid) ‡	7% 7% 85%	32% 17% 51%	N/A
**CSP Intra/post-procedure course**			
Intraprocedural bleeding (% cases)/endoclip assisted hemostasis (%)	7 (21) ***/3 (9)	9 (31)/9 (31)	16 (25)/12 (19)
2-week follow up	ED visit in 4 patients	ED visit in 2 patients	

CMH: Children’s Mercy Hospital, LPCH: Lucile Packard Children’s Hospital, CSP: cold snare polypectomy, ED: emergency department, HSP: hot snare polypectomy, HPS: hereditary polyposis syndrome, BBPS: Boston bowel preparation scale, pt: patient. * Procedure in which cecum was not intubated was notable for solid stool throughout the colon, preventing colonoscope advancement. ** Including piecemeal resection of larger lesions following saline lift. *** Unspecified if bleeding from CSP procedures/simultaneous hot snare polypectomy. † Complete info in 30/34 procedures ◊ Complete data in 30/34 procedures. ‡ Complete data available in 14/34 patients.

## Data Availability

The original contributions presented in the study are included in the article, further inquiries can be directed to the corresponding author.
